# Notch Signaling and Cross-Talk in Hypoxia: A Candidate Pathway for High-Altitude Adaptation

**DOI:** 10.3390/life12030437

**Published:** 2022-03-16

**Authors:** Katie A. O’Brien, Andrew J. Murray, Tatum S. Simonson

**Affiliations:** 1 Department of Physiology, Development and Neuroscience, University of Cambridge, Downing Street, Cambridge CB2 3EG, UK; ajm267@cam.ac.uk; 2 Division of Pulmonary, Critical Care and Sleep Medicine, University of California San Diego School of Medicine, La Jolla, CA 92093, USA

**Keywords:** hypobaric hypoxia, adaptation, Notch signaling, hypoxia-inducible factor

## Abstract

Hypoxia triggers complex inter- and intracellular signals that regulate tissue oxygen (O_2_) homeostasis, adjusting convective O_2_ delivery and utilization (i.e., metabolism). Human populations have been exposed to high-altitude hypoxia for thousands of years and, in doing so, have undergone natural selection of multiple gene regions supporting adaptive traits. Some of the strongest selection signals identified in highland populations emanate from hypoxia-inducible factor (HIF) pathway genes. The HIF pathway is a master regulator of the cellular hypoxic response, but it is not the only regulatory pathway under positive selection. For instance, regions linked to the highly conserved Notch signaling pathway are also top targets, and this pathway is likely to play essential roles that confer hypoxia tolerance. Here, we explored the importance of the Notch pathway in mediating the cellular hypoxic response. We assessed transcriptional regulation of the Notch pathway, including close cross-talk with HIF signaling, and its involvement in the mediation of angiogenesis, cellular metabolism, inflammation, and oxidative stress, relating these functions to generational hypoxia adaptation.

## 1. Introduction

Maintenance of an adequate oxygen (O_2_) supply is fundamental to cellular homeostasis and is challenged by impaired cellular O_2_ availability (hypoxia). Adjustments involving all points of the O_2_ cascade, from delivery to cellular usage, are crucial under hypoxic conditions, including disease states that impact the heart, lungs, and circulation across various life stages. Hypoxia can also be experienced upon exposure to high-altitudes, where barometric pressure falls and so the partial pressure of O_2_ falls. Despite this hypoxic stress, human populations have resided at altitudes above 3000 m in Tibet, Ethiopia, and the Andes for thousands of years. Over this time, natural selection of multiple gene regions and adaptive physiological traits has occurred [1,2,3,4]. Understanding the genetic variants under selection in these populations, and in other species with multigenerational exposure to hypobaric hypoxia, provides insight into mechanisms critical for tolerance and ultimately survival in hypoxia in all contexts [5]. 

The hypoxia-inducible factor (HIF) pathway is essential in the cellular hypoxic response. In normoxia, HIF-1α and HIF-2α are hydroxylated by the prolyl-hydroxylase (PHD) enzymes and so are targeted for ubiquitination by the von Hippel–Lindau (VHL) protein and degradation by the proteasome [6]. In conditions of low cellular PO_2_, HIF-1α/HIF-2α are stabilized, forming heterodimers with the nuclear-localized HIF-1β, and bind hypoxia response elements (HREs) in the promoter regions of hundreds of target genes [7,8]. Activation of the HIF pathway thus elicits a vast array of cellular responses in an O_2_-dependent manner. This includes genes that mediate O_2_ delivery, such as erythropoietin (*EPO*) and vascular endothelial growth factor (*VEGF*), which support O_2_ carriage and angiogenesis, respectively [9,10]. Beyond O_2_ delivery, the HIF pathway regulates cellular O_2_ utilization by targeting a plethora of metabolic genes, with the effect of enhancing glycolysis and lactate production [11] and attenuating fatty acid oxidation through suppression of peroxisome proliferator-activated receptor α (PPARα) [12,13]. 

HIF pathway genes play a key role in hypoxia adaptation in high-altitude populations. Genetic variation within the region of *EPAS1* (encoding HIF-2α) and *EGLN1* (encoding PHD2) have been identified across numerous studies of high-altitude adaptation [3,14,15], in addition to HIF-targeted genes such as *PPARA* [3] and non-HIF-related targets [2]. 

Whilst the HIF pathway is undoubtedly an essential mediator of the cellular response, adaptation to hypoxia is polygenic. The interplay of HIF with other genes known to be under selection, as well as those independent of the HIF pathway, are an important area under investigation. One such example is the highly conserved Notch signaling pathway, which functions in many different developmental and homeostatic processes, mediating a strikingly diverse range of downstream signals [14]. In this review, we provide a general overview of the Notch pathway and the studies that have identified *NOTCH* as a target of hypoxia adaptation. We further delve into the mechanisms by which the Notch pathway mediates cellular responses, and provide insight into its roles within the context of high-altitude adaptation.

## 2. Canonical Notch Signaling

Evolutionarily, Notch signaling is a highly conserved pathway that mediates communication between neighboring cells to regulate many developmental and homeostatic processes in metazoans. Upon ligand-mediated activation, Notch receptors form a complex that promotes transcription of target genes, leading to regulation of essential processes including cell proliferation and differentiation.

**Figure 1:**
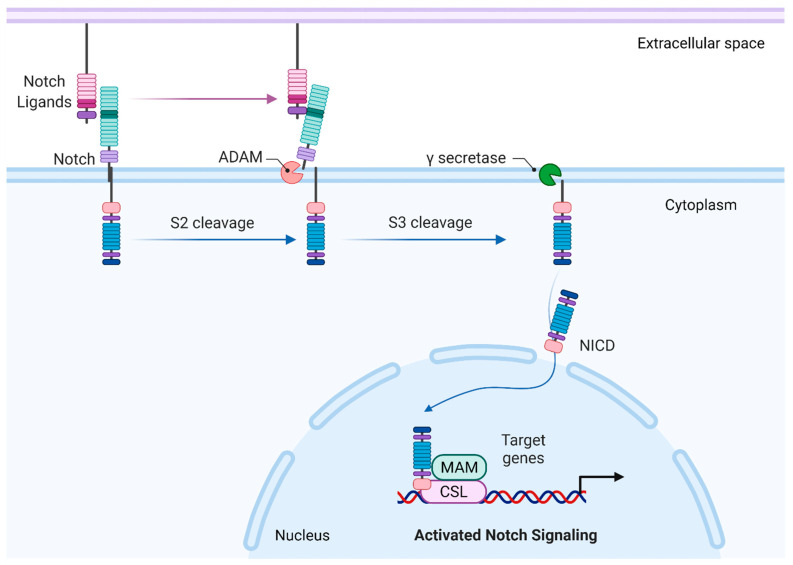
A simplified view of canonical Notch signaling. Summary of the core signaling pathway created with BioRender.com. Canonical Notch ligands bind to the Notch receptors at epidermal growth factor (EGF) repeats 11–12 (dark green sections). Cleavage of the Notch receptor involves two proteolytic cleavage events, the first catalyzed by ADAM metalloproteases at the negative regulatory region (purple sections), the second by γ-secretase. This releases the Notch intracellular domain (NICD). In the nucleus, NICD interacts with DNA binding protein CBF1/Suppressor of Hairless/LAG1 (CSL; also known as RBPJ) and the coactivator Mastermind (MAM) to promote gene transcription.

Notch receptors are large single-pass transmembrane proteins composed of an N-terminal extracellular subunit, a C-terminal transmembrane, and an intracellular subunit. Canonical transmembrane Notch ligands belong to the Delta–Serrate–LAG2 (DSL) family. Ligand binding to the extracellular region involves the DSL and amino-terminal domains, which contact epidermal growth factor (EGF)-like repeats 11–12 [15,16]. This exposes the cleavage site for S2 cleavage catalyzed by a disintegrin and metalloprotease (ADAM) family of metalloproteases, rendering the transmembrane-intracellular fragment as a substrate for S3 cleavage by the γ-secretase complex, which catalyzes intramembrane proteolysis, resulting in the release of the Notch intracellular domain (ICD). The Notch ICD comprises a membrane proximal RBP-Jk-associated molecule (RAM) region and an ankyrin repeat domain (ANK), both of which interact with the DNA-binding protein CBF1/Suppressor of Hairless/LAG1 (CSL; also known as RBPJ) and the coactivator Mastermind (MAM) to form a transcriptional complex that promotes the expression of target genes [17,18,19,20]. The Notch ICD also contains a nuclear localization sequence, located between the ANK and C-terminal proline/glutamic acid/serine/threonine-rich (PEST) domain. Termination of the active Notch ICD signal is mediated through ubiquitination of degron sites on the PEST domain and subsequent degradation by the proteasome [19]. A simplified overview of canonical Notch signaling is presented in Figure 1.

The output from canonical Notch signaling is enormously diverse and cell-context-dependent, operating in both physiological and pathological states. Activation of Notch signaling can promote cell growth and cancer development in some contexts, but cell death and tumor suppression in others [14]. Studies applying chromatin immunoprecipitation analysis provide insight into the wide array of genes regulated by Notch pathway signaling [21,22]. Notch ICD/CSL target genes include those related to cell-fate determination (*HES1* [23] and *HEY1* [24]), proliferation (*MYC* [25]), growth arrest (*p21* [26]), cancer stem cell markers (*CD44* [27], *BMI1* [28]), and Notch signal potentiation (*NOTCH3* [25]).

Despite this diversity in outputs, the cascade to activation and downstream signaling is relatively simple, involving the receptor–ligand interactions described to release the bioactive Notch ICD, with no intermediates from which signal amplification can occur. There is also limited diversity within the pathway components, with mammals possessing four Notch paralogues (Notch1–4) and various ligands in the Delta-like (DLL1, DLL3, and DLL4) and jagged pathways (Jagged1, Jagged2). *Drosophila melanogaster* possesses one Notch receptor and two ligands, the transmembrane proteins Delta and Serrate [14]. The plethora of downstream signals is therefore derived from regulatory mechanisms including ligand-expression patterns, pathway crosstalk, tissue topology, and the nuclear environment [14]. An example of a regulatory mechanism is the post-translational modification of the Notch receptor through addition of *N*-acetylglucosamine to *O*-fucose residues located in certain EGF repeats by Fringe family glycosyltransferases [29]. This modification alters relative affinity of ligand–Notch binding [30], leading to activation of Notch in response to Delta-like ligands, but suppression of downstream signaling for Serrate/Jagged ligands [31].

The cellular context for Notch signaling assessed in this review is hypoxia. Whilst interactions between the Notch pathway and the cellular hypoxic response are extensive [32], much of this interaction is related to cancer pathogenesis. Here, we considered Notch signaling in hypoxia through the lens of high-altitude adaptation. We begin by summarizing the evidence to show that genetic regions encoding Notch pathway proteins are under positive selection in hypoxia-adapted populations, and highlight research showing that this pathway is critical for conferring hypoxia tolerance.

## 3. Notch under Positive Selection

In humans, natural selection of Notch has taken place in highland native Andean and Tibetan populations. Investigations using locus-specific branch-length testing of single-nucleotide polymorphism (SNP) data have revealed positive selection for *NOTCH1*, alongside numerous HIF pathway candidate genes, in Andeans living above 3600 m [33]. Examination of the chromatin accessibility and transcriptional landscape in Tibetans residing at 3680 m, performed using human umbilical vein endothelial cells (HUVECs), identified two structural variants within the upstream regulatory element of *NOTCH1* (chr9:139420177-139429697, 67,694 bp) [34]. These variants, including a 200 bp duplication in chr9:139422831-139423031 and a 1200 bp duplication in chr9:139429296-139430493, were suggested to increase the accessibility of this regulatory element, and so to affect *NOTCH1* expression [34]. This finding was made alongside the identification of active regulatory elements cooperating to downregulate expression of *EPAS1*, encoding HIF-2α [34].

Positive selection of Notch signaling has also been identified in highland populations of some nonhuman species. For example, in Tibetan chickens, whole-genome resequencing identified *NOTCH2* as being under positive selection [35], whilst transcriptomic and proteomic analyses revealed enrichment of Notch signaling, specifically differential expression of *NOTCH2* [36]. In the Tibetan pig, transcriptome analysis revealed differential expression in *RBPJ (CSL)*, coding a crucial component of the Notch ICD transcriptional complex [37]. In a Tibetan yak, ADAM17 was identified as under selection [38]. NOTCH1 is a substrate for this metalloprotease, and it has been linked to regulation of ligand-independent Notch signaling [39]. The Notch1 pathway function is upregulated in an ADAM17-dependent manner in liver cancer stem cells [40]. However, its relevance in physiological conditions remains controversial, and unlike ADAM10, is reported to be dispensable for Notch activation [41].

These findings are in line with studies highlighting the importance of the Notch system in conferring hypoxia tolerance, a concept explored extensively in *Drosophila melanogaster*. Through generations of laboratory selection, a hypoxia-tolerant strain of *Drosophila* has been generated that is capable of survival in normally lethal O_2_ levels (4% O_2_) [42]. Examination of gene-expression profiles revealed upregulation of components in the Notch signaling pathway [42,43,44] alongside downregulation of genes encoding glycolytic, tricarboxylic acid (TCA) cycle, and β-oxidation enzymes [42]. The broad suppression of metabolic targets was coordinated by the Notch target transcriptional suppressor *hairy*, the mammalian homologue of which is *HES* (hairy and enhancer of split-1). Binding elements for *hairy* were located on the genes shown to be downregulated, with binding specifically occurring in hypoxia. Loss-of-function *hairy* mutants abolished the metabolic gene suppression, reducing hypoxia tolerance [42]. The importance of Notch signaling in this response was confirmed through application of a γ-secretase inhibitor, which significantly reduced survival and life span in hypoxia [44].

This evidence, summarized in Table 1 and Table 2, suggests Notch signaling is an important factor in hypoxic adaptation. However, the downstream effects of variants under positive selection at high-altitude are yet to be explored. To gain insight into potential avenues of interest when considering Notch genotype–phenotype interaction at high-altitude, we delved into the current understanding of Notch involvement in the cellular hypoxic response from the point of gene transcription, including interplay with the HIF pathway, to regulation of angiogenesis and vascular tone, metabolism, inflammation, and oxidative stress. The interplay of Notch with established hypoxia response pathways is summarized in Table 3.

## 4. Gene Transcription

### 4.1. Notch and HIF Cross-Talk

A multitude of evidence has demonstrated involvement of the Notch pathway in the transcriptional response to hypoxia, both up- and downstream of Notch ICD activation [32]. Upstream, the expression of Notch ligands Jagged2 [89,90] and Delta-like Ligand 4 (DLL4) [91] are upregulated in hypoxia. Downstream of Notch ICD activation, hypoxia induces expression of *NOTCH1* and targets such as *HES1*, as shown initially in human neuroblastoma cells cultured in 1% O_2_ [92]. Examination of mouse myogenic (C2C12) and neural precursor (P19) cells revealed a Notch1-dependent block on differentiation in hypoxia (1% O_2_)_,_ accompanied by the upregulation of Notch target genes *HES1* and *HES2* [46]. This effect was reversed with the use of an γ-secretase inhibitor, indicating Notch cleavage was essential for maintaining the undifferentiated cell state in hypoxia [46]. Direct interaction between HIF and Notch signaling was shown to be crucial in mediating this hypoxic cellular response.

An interaction interface between HIF-1α and Notch1 ICD was identified on the N-terminal region of HIF-1α, yet hypoxia-dependent activation of Notch signaling was shown to require the C-terminal region of HIF-1α [46]. Elsewhere in the Notch system, HIF-1α and HIF-2α interact with the promoters of Notch ligand *DLL4* and Notch targets *HEY1* and *HEY2* [46,47]. HIF-1α also interacts with the γ-secretase complex, leading to enhanced cleavage of the Notch ICD, and so Notch activation. The HIF- γ-secretase complex interaction occurs via the promoter region of subunit APH-1A in HeLa cells [48], but via the enzymatic component PS1 in breast cancer cells [49].

HIF–Notch interaction is bidirectional, as the Notch system has also been shown to mediate various components of the HIF pathway. Notch ICD activation increased recruitment of HIF-1α to the HRE-containing region of hypoxic-responsive genes *GLUT1*, *EPO*, and *VEGF* [45,46]. Notch ICD activation also upregulates HIF-2α at the transcriptional level, coinciding with increased expression of target genes *VEGF* and *AREG*, yet this interaction did not involve the HIF-2α proximal promoter [93]. In human medulloblastoma and breast cancer cell lines, the Notch-mediated upregulation of HIF-2α was accompanied by downregulation of HIF-1α, which the authors postulated may drive the potential transition from shorter-term HIF-1α supported responses to hypoxia to the more sustained responses characterized by HIF-2α signaling [93].

Cross-talk between the Notch and HIF pathways extends to regulators of HIF signaling, including interaction between factor inhibiting HIF (FIH)-1 with Notch ICD [46]. FIH is an asparaginyl hydroxylase that acts at residue 803 of HIF-1α to block HIF coactivator binding [50]. FIH-1 hydroxylates the Notch ICD at two conserved asparagine residues (N^1945^ and N^2012^) [45,94], binding with a higher affinity than it does to HIF-1α [45]. This binding negatively regulates Notch ICD activity, abrogating the Notch-mediated repression of neuronal and myogenic differentiation [45]. Interaction with FIH extends to other components of the Notch system, including E3 ubiquitin ligases Mindbomb 1 and 2 required for Notch activation [51,95], whilst in zebrafish, FIH mediates the antiangiogenic function of Mindbomb via VEGF-A [51]. Finally, an interaction was demonstrated between Notch 3 and the HIF pathway regulator VHL in breast carcinoma cells [96].

These studies indicated that the transcriptional response to hypoxia involves close interaction between the HIF and Notch pathways. The actions of both are therefore considered throughout the remainder of this review.

### 4.2. Epigenetic and Post-Transcriptional Modifications

In hypoxia, both epigenetic events and post-transcriptional modifications, including splice variation and the action of microRNAs, are crucial in the regulation of HIF and Notch signaling systems.

#### 4.2.1. Methylation

Epigenetic processes have been described as a central hub that connects environmental, physiological, and genomic inputs [97]. DNA methylation is an epigenetic modification that regulates chromatin organization and gene expression. The major site for DNA methylation in mammals is the cytosine-rich CpG dinucleotide, specifically at the 5′ position of the cytosine ring, mediated through the action of DNA methyltransferases (DNMTs). Whilst the vast majority of CpG sites across the human genome are methylated, interspersed among these are regions containing a high-density sequence of CpGs, or CpG islands, that often cluster within the promoter [98]. Hypomethylation of CpG islands most often enhances transcription-factor binding to activate gene expression, whereas hypermethylation often prohibits transcription-factor binding, leading to a quiescent chromatin state [99]. Global methylation status can be inferred through examination of long interspersed nuclear element-1 (LINE1) methylation, with decreased methylation being associated with genomic instability and cancer risk [100]. At high-altitude (above 4000 m), LINE1 methylation was greater in native Andean Quechua, compared with those of European ancestry [52,53]. In the lowlanders, this may be linked to increased reactive oxygen species (ROS) production (discussed below), as oxidative stress is associated with impaired methylation capacity [101].

Epigenetic silencing through methylation occurs in genes crucial to HIF expression and stabilization. An Andean Quechua resident at 4388 m showed decreased *EPAS1* methylation in comparison with those residing at sea level [52], whereas altitude exposure (4240 m) in subjects of European descent was associated with increased *EPAS1* methylation [53]. In line with the lowlander response, increased methylation of *EPAS1* has been observed in human renal tubule epithelial cells, occurring through the action of DNMT3a. This prevented the activation of HIF-2α and expression of downstream target genes, limiting the proliferative capacity of these differentiated cells under hypoxia [102]. Hypermethylation has also been observed on a CpG island within the 5′ region of *VHL* in human renal carcinoma, leading to inactivation [103].

Methylation affects the binding of HIF to its target genes, as the consensus core HRE contains a CpG dinucleotide, and upon methylation, HIF-1 DNA binding is abolished [104]. Indeed, HIF-induced expression of *EPO* is reliant upon a methylation-free HRE [104,105]. Methylation of CpGs within the *EPO* promoter impair transcriptional activation and block association of nuclear proteins, and this was suggested to act in concert with the CpG island in the 5′-untranslated region (UTR), which recruited methyl-CpG binding to the promoter, together silencing the *EPO* gene [106]. At high-altitude, *EPO* methylation decreased in subjects of European ancestry with ascent from 1400 m to 4240 m, thus facilitating increased expression in support of the erythropoietic response [53]. Alterations in other HIF targets included increased methylation of *PPARA* with increasing altitude, potentially suppressing fatty acid oxidation capacity [53].

Notch signaling is itself also closely regulated through DNA methylation [107]. Expression of *NOTCH1* and *NOTCH3* were methylation-dependent in hepatic satellite cells (HSC) [54], whilst the transcription factor downstream of the Notch receptor RBP-J (or CSL) binds DNA in a methylation-dependent manner [56]. DNA methylation also impacts Notch ligands, with hypermethylation of the *DLL1* promoter silencing expression in gastric cancer cells [55]. Treatment with a methylation inhibitor 5-aza-2′deoxycitidine resulted in increased DLL1 expression and activation of the Notch cascade, including the downstream target HES1 [55]. However, methylation is not always linked to expression of Notch pathway genes, as exemplified by *JAG2*, whereby methylation pattern of the promoter did not correlate with expression [108].

#### 4.2.2. Alternative Splicing

Alternative splicing of pre-mRNA increases transcriptomic and proteomic diversity by enabling the generation of multiple mRNA products from a single gene. Hypoxia is reported to drive intron retention above other splicing methods [109], and this may influence the fine tuning of both the HIF and Notch signaling systems.

Cassette exon skipping leads to several splice isoforms of HIF-1α [110,111,112], with the majority of isoforms conferring downregulation of HIF function [113]. An example is HIF-1α^736^, which lacks the C-terminal transactivation domain and displays a 3-fold lower activity than the full-length HIF-1α [111]. Conversely, HIF-1α^417^, which results from the skipping of exon 10, lacks a transactivation domain, yet promotes the nuclear translocation of HIF-1β, amplifying transcription of *EPO* [112]. Splice variation can also impact on factors interacting with HIF, such as peptidyl prolyl isomerase-1 (Pin1), a *cis/trans* isomerase that binds and stabilizes HIF-1α. Specifically, PIN1 transcript variant 2, identified as a long noncoding RNA, downregulated HIF-1α under hypoxia [114].

Multiple genes with characteristics of splice regulators display HIF1-α-dependent expression patterns, of which *C1QBP*, *HNRNPH3*, *JMJD6*, and *SF3B1* contain HREs within their promoters [115]. In myocardial hypoxia, regulation of splice factor 3b subunit 1 (SF3B1) by HIF-1α was shown to mediate the splicing of ketohexokinase (KHK) pre-mRNA, switching KHK-A for KHK-C. KHK-C displays a superior affinity for fructose, and this switch enforced fructolysis, a metabolic shift crucial for pathological growth [115]. Hypoxia-induced alternative splicing also impacts the regulation of glycolysis through HIF-1α-mediated splicing of glycolytic enzymes *Hk1* and *Pfkfb3* in myocardial hypoxia [115], as well as differential regulation of lactate dehydrogenase (*LDHA*) variants through intron retention in breast cancer cells [109]. In the context of both cancer [116] and myocardial infarction [117], a switch in the expression of pyruvate kinase isoforms occurs, with the embryonic isoform PKM2 being re-expressed above the level of the adult isoform PKM1. This was mediated through RNA binding proteins and sequence-specific splicing repressors hnRNAP1 and hnRNAP2, and was linked to the promotion of aerobic glycolysis [116,117]. PKM2 acts in a positive feedback loop through interaction with PHD-3 to promote HIF-1α transactivation and reprogram glucose metabolism [118].

Alternative splicing of components of the Notch signaling system has been investigated extensively in the context of chronic lymphocytic leukemia (CLL), where Notch1 and 2, along with ligands Jagged1 and 2, are constitutively expressed [119]. Recurrent ‘noncoding’ mutations were clustered at the 3′-UTR region of *NOTCH1* in ~2% of patients with CLL or monoclonal B-cell lymphocytosis, with the most common being located at chr9:139390152 [120,121]. These mutations were characterized by within-exon splicing, and were predicted to remove the PEST domain of Notch1, constitutively activating downstream signaling [120]. In some instances, noncoding variants occurred alongside mutations in the splicing factor SF3B1 [121], which increased Notch signaling through an alternate splice variant of the Wnt pathway member DVL2 [122].

#### 4.2.3. MicroRNA

MicroRNAs (miRs) are a class of noncoding RNAs with a functional ~20–22 nt sequence that targets mRNAs, thereby inducing translational repression or RNA degradation [123]. Again, this method of modification interacts with both the HIF and Notch signaling systems.

HIF-1α enhances the induction of miR-210, the targets of which include regulators of mitochondrial respiratory function, as shown through alterations in placental metabolism [76], DNA repair, cell survival, and angiogenesis [124]. On the latter, induction of miR-210 was linked to upregulation of the Notch1 protein, and in turn to stimulation of angiogenesis following cerebral ischemia [125]. In the trophoblast, miR-210 represses cytoplasmic polyadenylation element binding 2 (CPEB2), which is a negative regulator of HIF-1α translation, thus forming a positive feedback loop of HIF-1α induction [126].

Encoded within intron 4 of the Notch1 locus is miR-4673. Transcription of miR-4673 altered cell cycle function in breast cancer [127] and neurogenic [128] cells, involving indirect regulation of Notch function alongside β-catenin and p53, and this has been linked to loss of mitochondrial membrane potential and ROS generation in human carcinoma cells through targeting 8-oxoguanine DNA glycosylase 1 [129]. Proximal to the Notch1 locus is miR-4674, which regulates angiogenesis through interaction with p38 VEGF signaling in endothelial cells [130].

Cross-talk between epigenetic and post-transcriptional elements has also been noted. For instance, methylation of miR-34a was linked to the expression of Notch pathway genes (*NOTCH1*, *NOTCH2*, and *JAG1*) in cholangiocarcinoma cells [131], whilst changes in methylation pattern are known to alter inclusion levels of alternatively spiced exons [132].

The evidence thus suggests that the Notch pathway plays a key role in the regulation of transcriptional response to hypoxia at multiple levels. In addition, the Notch pathway is known to mediate numerous processes linked to cellular and tissue remodeling upon high-altitude exposure, including regulation of angiogenesis and vascular tone, metabolism, inflammation, and oxidative stress.

## 5. Hypoxic-Induced Cellular and Tissue Remodeling

### 5.1. O_2_ Delivery

#### 5.1.1. Erythropoiesis

In most nonadapted individuals, high-altitude exposure induces the generation of red blood cells from hematopoietic stem cells (HSCs); i.e., erythropoiesis. Whilst this response increases O_2_ carriage capacity, in excess it can result in high blood viscosity. This impairs tissue blood flow and O_2_ delivery, and is a feature of chronic mountain sickness (CMS) [133]. In native highlanders, the erythropoietic response is variable. Andeans display elevated hemoglobin and hematocrit alongside a high prevalence for CMS [134,135]. The majority of Tibetan highlanders, however, maintain hemoglobin and hematocrit at levels comparable to those of populations living at sea level, and lower than many of their Andean counterparts for any given altitude [134,136]. In line with this, Tibetans present lower blood oxygen saturations [1] and are protected from the detrimental effects of polycythemia and a lower prevalence of CMS [135].

An essential mediator of erythropoiesis in hypoxia is the increased production of *EPO* from kidney interstitial cells, which binds erythroid progenitors in bone marrow, stimulating their survival, proliferation, and differentiation. HIFs directly regulate *EPO* expression and also impact iron metabolism in hypoxia [137,138]. In the Tibetan population, the decreased hemoglobin phenotype has been linked to a variant in *EGLN1*, which promotes HIF degradation [3,139], as well as putatively adaptive copies of *EPAS1* and *PPARA* loci [3], the latter being a HIF target. However, HIF is not the only regulator of erythropoiesis, which is a multistage process mediated through complex intracellular networks involving coordinated gene expression by transcription factors, chromatin modifiers, and miRNAs [140]. Genes related to different stages of erythropoiesis have been identified in highland populations [141], such as *SENP1*, which has been associated with development of CMS in Andeans [142,143].

Notch signaling is crucial for regulating stem cells in various settings [144], including embryonic hematopoiesis [145]. For example, disruption of *NOTCH1* and downstream signaling in the mouse embryo was shown to impair HSC formation [146,147]. However, the involvement of Notch signaling in HSC homeostasis post development is less clear. Multiple reports from gain-of-function experiments demonstrate increased Notch signaling enhanced self-renewal of HSCs [148]. Notch is also expressed in human bone marrow CD34^+^ hematopoietic precursors [149], and there is interplay between Notch and HSC regulatory factors, including parathyroid hormone and stem cell factor (SCF). Parathyroid hormone increased the levels of *Jagged1* in the bone marrow, and hematopoietic cell growth was abrogated through γ-secretase inhibition [150]. SCF-induced expression of *Notch2* and SCF-mediated expansion of primary erythroid precursors were linked to Jagged1 [151]. Blocking Notch2 signaling inhibited the proliferative effects of SCF [151]. However, evidence also implies Notch activity is dispensable in the maintenance of HSCs, as shown through blockage of canonical HSCs in adult bone marrow [152] and through low levels of Notch receptors in purified HSCs [153]. The relevance of Notch signaling in the regulation of erythropoiesis at high-altitude has not been explored, but warrants further investigation, particularly given the role of Notch in maintaining cells in the stem/progenitor state in hypoxia [46].

#### 5.1.2. Angiogenesis

Adequate tissue perfusion is critical for maintaining O_2_ homeostasis. Adaptation of the vascular system to hypoxia includes angiogenesis, a process requiring upregulation of proangiogenic signals to mediate each stage of growth, from extracellular matrix re-modelling to tube formation [154]. Examination of native highland human populations and other species has shown angiogenic pathway genes to be under positive selection at high-altitude. For instance, gene network analysis revealed selection of integrin subnetworks in Tibetan and Sherpa populations [155], whilst assessment of SNP data revealed selection for *VEGF* in Andean populations [33]. In the Tibetan chicken, whole genome re-sequencing and comparative transcriptomic and proteomic analyses revealed strong selection for *VEGF* [35,36], whilst genome wide analysis of the Tibetan sheep identified selection of genes within the Ras/ERK signaling pathway [156]. At a tissue-specific level, assessment of protein expression in placentas of Andean pregnancies revealed lower levels of the antiangiogenic factor sFlt-1 (soluble fms-like tyrosine kinase) and a lower sFlt-1/placental growth factor (PLGF) ratio, alongside higher uterine artery blood flow and birthweight compared with European pregnancies at the same altitude (3600 m) [157]. This is in line with findings demonstrating graduated protection against reduced birthweight in high-altitude pregnancies in Andean mothers compared to those with European ancestry [158].

A multitude of proangiogenic factors have been identified as HIF targets, including VEGF, PLGF, and integrin [57]. HIF regulation of hypoxic proangiogenic signals has been implicated during development and both adaptive physiological and pathological states [57]. Notch signaling is also a key mediator of angiogenesis at all life stages, with disruption to this pathway impacting trophoblast invasion [159] and embryo development [160] through mature tissue function [161]. Angiogenic mediation via the Notch system has, to date, largely focused upon interplay with VEGF and the effects on vascular integrity.

The initiation of blood vessel branching during angiogenesis, termed sprouting, requires coordination of endothelial cell behavior with selection of a filopodia-rich leading tip cell and following stalk cells. This process is mediated through a tight interplay between the VEGF-A receptor VEGFR, the Notch ligands DLL4 and Jagged, and downstream Notch signaling [59,162]. Key to the selection of tip and stalk cells are differential *VEGFR1* and *VEGFR2* levels mediated via DLL4 [59]. High levels of DLL4 in endothelial tip cells activate Notch in adjacent stalk cells to downregulate *VEGFR* expression, suppressing the tip phenotype and instead enabling formation of the stable vasculature [163,164]. DLL4–Notch signaling is antagonized by Jagged1, which competes with DLL4 binding in cells expressing Fringe family glycosyltransferases, promoting endothelial sprouting and tip formation [58].

The Notch system is therefore critical for mediation of vascular integrity and homeostasis. Indeed, gene inactivation of either Notch1 or DLL4 is embryonic lethal due to deregulation of angiogenesis and the loss of artery–vein specification [160,165]. Notch signaling is also critical to the regulation of vascular function in numerous pathologies characterized by tissue hypoxia, including tumor angiogenesis [166]. Blockade of Notch/DLL4 enhanced hypoxia in mouse subcutaneous C6 tumors [167], and decreased tumor growth [167,168]. However, the effect of Notch blockade on tumor development is context-dependent, as activation of the Notch system in endothelial cells promotes metastasis in the lung [169], but represses metastasis in the liver [170].

To add to the complexity of angiogenic regulation, VEGF-A, alongside its receptors VEGFR1 and VEGFR2, exhibit splice variation, with differing effects on bioavailability, binding capacity, and downstream signaling [113,171]. For instance, activation of VEGF-A_121_ induced a reduced rate of VEGFR2 phosphorylation, resulting in impaired motility and sprouting in human endothelial cells [172]. Alternative splicing can also result in soluble isoforms of VEGFR1, which are largely similar through exon 13, but differ in the C-terminus, including sVEGFR1-i13. Regulation of sVEGFR1-i13 involves splicing factors SRSF2 [173] and U2AF65 [174] alongside the NOTCH1 signaling system, with differential regulation exhibited by the Jagged and DLL ligands [175].

#### 5.1.3. Vascular Tone

Hypoxic exposure can lead to onset of pulmonary hypertension (PH). This disorder is prevalent in high-altitude populations [176], although evidence suggests Tibetans may be protected by displaying a lack of muscularization of pulmonary arteries and low hypoxic pulmonary vasoconstriction [177]. Notch signaling has been linked to the onset of this disorder, specifically via Notch3. Expression of Notch3 mRNA and Notch3 ICD were increased in lung tissue obtained from PH patients, mice with hypoxia-induced PH, and rats with monocrotaline-induced PH [61]. In mice, PH was prevented via homozygous deletion of Notch3 and administration of a γ-secretase inhibitor [61]. Expression of both Notch3 and target Hes-5 were confined to vascular smooth muscles within small pulmonary arteries, and were required for cell proliferation and development [61]. Notch3 signaling was associated with increased vascular resistance, characteristic of PH, through regulation of intracellular Ca^2+^, achieved via enhancing store-operated Ca^2+^ entry through upregulation of canonical transient receptor potential (TRPC) channels [63] and increased expression of the Ca^2+^-sending receptor [64].

Another pathology associated with vascular control and Notch signaling is pre-eclampsia, a major cause of maternal and fetal morbidity and mortality [178] that increases in prevalence at high-altitude [179,180]. This multisystem pregnancy disorder is characterized by hypertension, proteinuria, and placental hypoxia and dysfunction that is attributed to the shallow implantation and defective spiral artery remodeling [181]. Specifically, HIF-1α promotes invasion of human trophoblast cells and angiogenesis via Notch1, with upregulation of proangiogenic factors including endothelin receptor type B (ETBR) via activator of transcription 3 (STAT3) and VEGF [159]. This signaling was disrupted in patients with pre-eclampsia, with downregulation of placental HIF-1α, Notch1, and ETBR [159].

### 5.2. Cellular Metabolism

Hypoxia is a particular stressor on metabolic homeostasis, and specifically oxidative metabolism. In hypoxia, metabolic adjustments are required to sustain mitochondrial ATP production in the face of a reduced O_2_ supply and increased oxidative stress [12,65]. Metabolic remodeling is thus an essential component of the hypoxic cellular response [78,182]. This can be observed through proteomic [183,184] and metabolomic [12,166,168] analyses, which have demonstrated significant remodeling of metabolic pathways and tissue metabolites upon ascent to high-altitude.

Metabolic remodeling in the face of hypoxic exposure broadly includes modulation of insulin sensitivity, glycolysis, mitochondrial oxidative phosphorylation, and lipid oxidation. HIF is a well-established regulator of the metabolic hypoxic response, acting as a metabolic switch to optimize mitochondrial respiratory function [68,185]. Notch signaling is also a key modulator of metabolic homeostasis [186], with activation being associated with raised mitochondrial membrane potential alongside increased ATP/ADP and NADH/NAD ratios [72]. Whilst much of the evidence supporting this has been obtained in normoxia or in the context of cancer, it involves a multitude of metabolic targets and systems known to influence metabolic remodeling at high-altitude.

#### 5.2.1. Glucose Homeostasis and Insulin Sensitivity

Hypoxic exposure impacts glucose handling via modulation of insulin sensitivity, which is in turn influenced by factors including ethnicity [187] and pathologies associated with nutrient excess and chronic inflammation [188]. The response in hypoxia appears to be dependent on degree and duration of exposure. Loss of insulin sensitivity has been reported with prolonged exposure to extreme altitude [65,66] and intermittent hypoxia [189], whereas shorter duration of moderate hypoxia improved insulin sensitivity in obese subjects [190]. Notch signaling has been linked to regulation of glucose uptake by liver and adipose tissue, with overactivation of the Notch system impairing insulin sensitivity [67,75,79]. In the liver, Notch signaling occurred in concordance with the transcription factor forkhead box protein O1 (FoxO1) via the Notch ICD, with Notch1 gain-of-function promoting insulin resistance and glucose-6-phosphatase expression in a FoxO1-dependent manner [67]. Notch activity in endothelial cells has been linked to the regulation of muscle insulin uptake via caveolae genes, with sustained Notch signaling lowering insulin sensitivity and increasing blood glucose; however, inhibition resulted in improved insulin sensitivity and improved glucose regulation [191].

#### 5.2.2. Glycolysis

Downstream of glucose uptake, hypoxia is commonly reported to enhance anaerobic glycolysis and increase the production of extracellular lactate, which can itself be transported into cells for use as metabolic substrate. This effect has been observed upon high-altitude exposure in lowlanders [192], whilst enhanced capacity for lactate production was observed in Sherpa skeletal muscle through higher lactate dehydrogenase (LDH) enzyme activity [12]. Increased reliance upon glycolysis is a common observation in hypoxic animal models [193] and in pathological contexts, such as tumors or ischemic injury [194]. HIF mediates this response through upregulating genes that encode glycolytic enzymes [11], alongside a shunting of pyruvate toward lactate production through the upregulation of pyruvate dehydrogenase kinase [68,185]. Notch also mediates glycolytic function through two distinct mechanisms. The first involves hyperactivated Notch signaling via the phosphatidylinositol 3-kinase (PI3K)/AKT serine/threonine kinase pathway to upregulate glycolytic targets such as the enzyme hexokinase and glucose transporter 1, as seen in breast cancer cells [69] and *Drosophila* [70]. The second mechanism involves hypoactivated Notch signaling, leading to reduced p53 levels, which enhanced glucose uptake at the expense of suppressed mitochondrial activity [32]. The ability of the Notch pathway to act as a metabolic switch for glucose flux has further been demonstrated in macrophages, in which M1 macrophage activation was reliant on Notch1-dependent induction of pyruvate dehydrogenase phosphatase 1 expression, pyruvate dehydrogenase activity, and glucose flux into the TCA cycle [195].

#### 5.2.3. Glutamine Metabolism

In hypoxia, the shift toward greater lactate production leads to the lack of reduced carbon for a functional electron transport chain, fatty acid synthesis, and ultimately cell proliferation [196]. An alternative carbon source is therefore sought in the form of glutamine [196,197]. Through an HIF-2α-dependent mechanism, reductive metabolism of glutamine to the TCA cycle intermediate α-ketoglutarate is redirected toward de novo lipogenesis via formation of the fatty acid palmitate [71]. This pathway has been implicated in high-altitude exposure, with increased expression of skeletal muscle glutamine synthetase [184]. Through proteomic analysis, activation of the Notch system was associated with decreased glutamine consumption alongside downregulation of three glutamate catabolism enzymes: glutaminase, ornithine aminotransferase, and glutamate dehydrogenase 1 [72].

#### 5.2.4. Mitochondrial Network and Respiration

Suppression of mitochondrial function with hypoxic exposure can occur through multiple mechanisms, including regulation of the mitochondrial network. For instance, expression of mitochondrial biogenesis factor peroxisome proliferator-activated receptor gamma coactivator 1 alpha (PCG1α) declined in human skeletal muscle following prolonged exposure to extreme high-altitude [74], occurring alongside a loss of muscle mitochondrial volume density, particularly within the subsarcolemmal population [74,198]. PGC1α is mediated both by HIF [73] and also Notch, as the proximal promoter region of PGC1α contains a binding site for the Notch target HES1. Binding of *Hes1* suppressed *Pgc1α* expression in mouse adipocytes, whilst deficiency of Notch1 led to elevated Pgc1α protein levels [75].

Specific alterations of the respiratory chain induced by hypoxic exposure include suppression of complex I, demonstrated in hypoxic rat heart [199], human skeletal muscle [184], and placenta [76]; whilst complex IV is altered through an HIF-mediated switch of subunits to optimize respiratory efficiency [77]. The Notch system should be considered in this context, as hypoactive Notch induces a decrease in complex I activity and in expression of the complex IV subunit COXII through p53 signaling in breast cancer [32]. Conversely, Notch1 hyperactivation has also been associated with downregulation of complex I subunits NDUFS1 and NDUFV2, as assessed through proteomic analysis of human immortalized leukemia cells (K562) [72].

##### 5.2.5. β-Oxidation

Hypoxic exposure is associated with suppressed β-oxidation capacity [12,193], a response modulated by HIF through repression of the fatty-acid-activated transcription factor peroxisome proliferator-activated receptor α (PPARα) [12,78,194], with changes to its downstream targets including the mitochondrial transporter carnitine palmitoyl transferase (CPT) [12,193]. A putatively advantageous *PPARA* haplotype has been identified in Tibetans [3], and this was associated with metabolic adaptation in Sherpas at high-altitude [12].

Notch signaling has profound effects on the regulation of β-oxidation, although these are likely to be cell-context dependent. Notch1 deficiency has been associated with increased expression of *Ppara* and *Cpt1* in liver [79] and adipose tissue [75]. Disruption of Notch signaling has been linked to browning of white adipose tissue, with higher metabolic rate dependent on increased uncoupling protein 1 expression and resistance to obesity induced by a high-fat diet [75]. Similarly in the liver, abrogated Notch signaling was associated with enhanced β-oxidation capacity, resulting in decreased hepatic lipid accumulation [79]. Notch signaling is positively correlated with fatty liver disease [200] and stimulation of lipogenesis has been linked to Notch-dependent stabilization of mammalian target of rapamycin complex 1 (mTORC1) [84]. The interaction with mTORC1 is bidirectional, as mTORC1 stimulated via excess amino acid consumption upregulates Notch1 signaling through STAT3 [201]. In leukemia cells, Notch activation was associated with increased levels of the β-oxidation enzyme mitochondrial enoyl-CoA hydratase [72].

### 5.3. Inflammation

Tissue hypoxia is known to augment inflammatory signaling. For instance, sustained exposure of lowlanders to high-altitude (6–8 weeks, >5300 m) led to a sharp rise in circulating interleukin (IL)-6, a target of the immune system regulator nuclear factor (NF)-kB [65]. NF-kB is of particular interest given its cross-talk with HIF [202]. This interaction involves amplification of NF-kB signaling through HIF binding Toll-like receptors [80] and enhanced HIF transcriptional activity through direct binding of the NF-kB precursor IkBα to FIH [81]. ROS-mediated upregulation of HIF-1α was shown to be dependent on NF-kB, and transfection experiments revealed an unidentified NF-kB binding element on the HIF-1α promoter [82]. HIF–NF-kB cross-talk has been implicated in diseases in which chronic hypoxia occurs concurrently with chronic inflammation, including cancer [203].

Regulation of NF-kB also involves complex cross-talk with Notch signaling at multiple points of each pathway [204,205], with physical interaction between the N-terminal portion of Notch ICD and NF-kB subunit p50 [83]. Cooperation of Notch with other immune system regulators includes interaction between Notch1 and transforming growth factor-β (TGFβ) in the maintenance of regulatory T cells through the transcription factor Foxp3 [206]. Indeed, Notch signaling is an important regulator of both the innate and adaptive immune responses and regulates immune cell development and function. This includes determination of cell lineage in developing lymphocytes and regulation of T- and B-cell differentiation and function [207], with Notch inactivation leading to accumulation of B cells in the thymus and blocking of T-cell development [206,208].

As with all aspects of Notch signaling, Notch regulation of immune system function is context-dependent [205]. In endothelial cells, Notch1 ICD activity in human carcinomas and melanoma orchestrated tumor progression and metastasis through increasing expression of chemokines and the adhesion molecule VCAM1, which promotes neutrophil infiltration and tumor cell adhesion [169]. In luminal endothelial cells derived from the aorta of coronary artery disease patients, upregulation of Notch pathway components at atherosclerotic lesions resulted in upregulation of inflammatory signaling, including IL-6 and ICAM1 and induction of endothelial cell senescence [209].

### 5.4. Oxidative Stress

ROS production is a key element of the cellular hypoxic response, involving multiple cellular sources, including mitochondrial complexes I and III. ROS signaling affects a multitude of downstream pathways, including HIF-1α, NF-kB, and nuclear factor erythroid-2 related factor 2 (Nrf2), with the latter being a regulator of genes that modulate oxidative stress, including *GSTA2* and *NQO1* [85,86,210,211]. An imbalance between ROS production and antioxidant capacity leads to oxidative stress, resulting in production of lipid and protein oxidation products. Markers of oxidative stress have been reported in lowland subjects ascending to high-altitude, leading to increased circulating isoprostanes [65] and increased skeletal muscle oxidized/reduced glutathione and sulfoxide/total methionine [12]. Of note, no such change was observed in an adapted Sherpa population following the same ascent profile [12]. Increased oxidative stress has been demonstrated in Andeans at high-altitude compared to lowlanders in normoxia [212,213]. This included elevated levels of circulating ascorbate free radicals alongside depressed antioxidants, an effect that was more pronounced in sufferers of chronic mountain sickness [212,213].

Notch signaling is an important modulator of oxidative stress. In the rat heart, Notch1 signaling via Nrf2 decreased ROS production and increased antioxidant activities to exert myocardial protection in ischemia–reperfusion injury [87]. Myocardial Notch signaling also acts via the Janus kinase 2 (JAK2)/signal transducer and STAT3 to activate mitochondrial superoxide dismutase expression and decrease ROS production [88]. In hepatocytes, Notch–STAT3 cross-talk has also been demonstrated, again in the context of ischemia–reperfusion injury, attributed to HES5-dependent STAT3 activation [214]. Conversely, ROS production has been linked to the regulation of Notch signaling function via NADPH oxidase 1, impacting cell proliferation and postmitotic differentiation [215].

## 6. Future Directions

Further investigation into the downstream effects of the specific *NOTCH* variants under selection in highland populations could reveal novel insights into mechanisms crucial to adaptation to hypoxia. A vast range of methodology exists to enable identification of specific variants and investigation into functional mechanisms [216]. In the identification of variants under selection, whole-genome sequencing provides the opportunity to obtain greater resolution of large-scale genome datasets alongside insight into noncoding regions. The functional mechanisms of Notch signaling in hypoxia are clearly complex, from the point of transcriptional regulation to a multitude of downstream pathways. Application of systemwide omics techniques would enable the molecular intricacies of this cross-talk to be captured, from the transcriptome and epigenome to the proteome and metabolome [192,217]. The application of these approaches in human subjects is crucial for providing insight into the genotype–phenotype association, whilst insight into direct causal links can be probed through genome-editing technologies [216].

## 7. Conclusions

Positively selected genetic regions that include Notch pathway components have been identified in highland populations, whilst Notch signaling has also been attributed to hypoxia tolerance. Together, this suggests that the Notch pathway is a key player in the complex interaction of regulatory pathways mediating hypoxic adaptation. The implications of Notch genetic variants under selection at high-altitude are, however, largely unexplored and warrant further investigation. Crucially, the Notch system exhibits close cross-talk with multiple elements of the HIF pathway, and is known to mediate numerous processes that are linked to cellular and tissue remodeling upon high-altitude exposure. Unravelling the intricacies of the high-altitude-selected Notch variant genotype–phenotype interaction will thus require a multisystem approach that spans from the transcriptome to the proteome and metabolome.

## Figures and Tables

**Table 1:** Notch under selection at high-altitude.

Subjects/Species	Highland/Hypoxic Location	Altitude (m)	Data Format	Test for Natural Selection	Positively Selected Notch Gene/Region	Reference
Human, Andeans (*n* = 50)	Cerro de Pasco, Peru (Quechua) La Paz, Bolivia (Aymara)	4300 3600	SNP genotype, Affymetrix, Inc. Gene Chip Human mapping 500 k array	Locus-specific branch length for SNP loci	*NOTCH1*	[33]
Human, Tibetans (*n* = 131)	Lhasa, Tibet	3680	Chromatin accessibility landscape through paired ATAC-seq and RNA-seq, obtained from primary HUVECs	Variant interpretation model by paired expression and chromatin accessibility methodology to identify active selected regulatory elements	*NOTCH1* regulatory element	[34]
Chicken, Tibetan (*n* = 9)	Xiangcheng County, Tibet	3500	Whole genome resequencing	Aligned to reference genome using SOAP2, detected short InDel and structure variants	*NOTCH2*	[35]
Pig, Tibetan (*n* = 2)	Diqing Tibetan autonomous prefecture, Yunnan province	3500	Lung tissue whole-transcriptome microarrays	Differential gene expression, regulatory and phenotypic impact factor analysis	*RBPJ (CSL)*	[37]
Yak, Tibetan (*n* = 1)	Huangyuan County, Qinghai province	3700	Short oligonucleotide analysis	Whole-genome shotgun assembly	*ADAM17*	[38]

**Table 2:** Notch involvement in hypoxia tolerance in laboratory experiments.

Species	%O_2_	Duration of Hypoxia Exposure	Data Format	Test for Natural Selection	Positively Selected Notch Gene/Region	Reference
Drosophila (*n* = 100)	5% O_2_	1 week	P-element insertion line screen	Genomewide screening of P-elements related to eclosion rate, rtPCR on selected P-element targets	*Dip1*, *CG14782*, *mRpS18B*, *Mys45A*, *CG6230*, *Drp1*, *Rep2*, *osa*, *CG8116*, *Atg1*, *CG33169*, *Chro*, *pzg*, *polo*, *lqf*, *Scrib*, *Alh*, *tna*, *CG14185*, *ci*	[43]
Drosophila (*n* = 200, derived from 27 parental strains)	6% and 4% O_2_	3 weeks for Notch mutants	Whole genome re-sequencing	Aligned to reference genome using MAQ, identified loci with high-confidence allelic differences and regions with allelic frequency differences	Fixed SNPs/indels: *Notch*, *Delta*, *Fringe*, *Sgg* *Hairless*, *HDAC4*, *Fur2*, *Bon*, *IP3K2*, *Nej*, *Pcaf*, Change in gene regulation: *E(spl) Cluster Genes*, *Aph-1*, *Nct*, *Ser.*	[44]
Chicken, Tibetan (*n* = 9)	13% O_2_	11 days	Transcriptomic and proteomic analysis of embryos	Differentially expressed protein.	*NOTCH2*	[36]

**Table 3:** Notch involvement in physiological responses to hypoxic exposure.

Hypoxic Physiological Response	Related Signaling Factors	References	Notch Pathway Interplay	Effect of Notch Interplay	Model/Condition	References
Modification of gene transcription	HIF-1α, HIF-2α	Reviewed in [6]	Notch ICD	Recruitment of HIF to HREs	Mouse myogenic and embryonic teratocarcinoma cells, hypoxia (1% O_2_)	[45,46]
			DLL4, HEY2	Notch pathway activation	Mouse myogenic and embryonic teratocarcinoma cells and human epithelial and embryonic kidney cells, hypoxia (1% O_2_)	[46,47]
			γ-Secretase complex	Enhanced cleavage of Notch ICD	Human epithelial and breast cancer cells, hypoxia (NiCl_2_ and 1% O_2_)	[48,49]
	Factor inhibiting HIF (FIH)	[50]	Notch ICD	Cellular differentiation	Mouse myogenic and embryonic teratocarcinoma cells, hypoxia (1% O_2_)	[45,46]
			Mindbomb 1 and 2	Angiogenesis	Zebrafish embryos	[51]
	DNA methylation	[52,53]	*NOTCH1*, *NOTCH3*, *DLL1*	Notch pathway expression	Rat hepatic stellate cells and human gastric cancer cells	[54,55]
			CSL (or RBP-J)	Methylation-dependent DNA binding	Human leukemia cells	[56]
Angiogenesis	HIF via VEGF	Reviewed in [57]	Notch via DLL4 and Jagged1	Differential VEGFR expression for selection of tip and stalk cells	Mouse embryo	[58,59]
Increased vascular tone	Pulmonary vascular remodeling	[60]	Notch3 pathway	Smooth muscle cell proliferation in small pulmonary arteries	Human and rodent pulmonary hypertension	[61]
	Increased intracellular Ca^2+^	Reviewed in [62]	Notch3 pathway	Upregulation of TPRC channels and increased expression of the Ca^2+^-sending receptor		[63,64]
Loss of insulin sensitivity	Increased plasma glucose and insulin	[65,66]	Notch ICD via FoxO1	Insulin resistance, increased glucose-6 phosphatase expression	Mouse liver, normoxia	[67]
Upregulated glycolysis	HIF via PDK	[68]	Notch via PI3K/AKT serine/threonine kinase	Increased glucose uptake, and upregulation of glycolytic genes	Human breast cancer cells and Drosophila	[69,70]
			Notch via p53	Glycolytic dependency, suppressed mitochondrial activity	Human breast cancer cells	[69]
Glutamine metabolism	HIF-2α	[71]	Notch1 pathway	Decreased glutamine consumption and expression of glutaminase, ornithine aminotransferase and glutamine dehydrogenase 1	Human immortalized leukemia cells and T lymphocytes	[72]
Loss in mitochondrial density	HIF via PGC1α	[73,74]	HES1	Suppressed PGC1α	Mouse adipocytes	[75]
Suppressed respiratory complex I	HIF via mir-210	[76]	Notch1 pathway	Decreased complex I activity and subunit expression	Breast cancer and immortalized leukemia cells	[72]
Complex IV subunit switch	HIF	[77]	Notch1 via p53	Downregulated CIV	Breast cancer	[32]
Suppression of β-oxidation	HIF, PPARα	[3,12,78]	Notch1 pathway	Notch1 pathway inhibition increased *PPARA* and *CPT1* expression	Mouse models of Notch deficiency, liver and adipose	[75,79]
Inflammation	HIF, NF-kB	[65,80,81,82]	Notch ICD	Interaction with NF-kB subunit	Human T cells	[83]
Oxidative stress	ROS via HIF, NF-kB, Nrf2	[84,85,86]	Notch1 via Nrf2	Increased cell viability, reduced ROS formation, increased antioxidant activities	Neonate rat myocardial cells, hypoxia–reoxygenation	[87]
			Notch1 via JAK2/STAT3	Activated mitochondrial SOD expression and decreased ROS production	Rat myocardium, burn injury	[88]
